# Modeling the effect of the cephalic phase of insulin secretion on glucose metabolism

**DOI:** 10.1007/s11517-019-01950-x

**Published:** 2019-01-26

**Authors:** Amparo Güemes, Pau Herrero, Jorge Bondia, Pantelis Georgiou

**Affiliations:** 1 Centre for Bio-Inspired Technology, Department of Electrical and Electronic Engineering, Imperial College London, South Kensington Campus, London, UK; 2 Instituto Universitario de Automática e Informática Industrial, Universitat Politècnica de València, Valencia, Spain; 3 Centro de Investigación Biomédica en Red de Diabetes y Enfermedades Metabólicas Asociadas (CIBERDEM), Madrid, Spain

**Keywords:** Cephalic phase, Neural modeling, Insulin secretion, Vagus nerve, Metabolic modeling

## Abstract

The nervous system has a significant impact in glucose homeostasis and endocrine pancreatic secretion in humans, especially during the cephalic phase of insulin release (CPIR); that is, before a meal is absorbed. However, the underlying mechanisms of this neural-pancreatic interaction are not well understood and therefore often neglected, despite their significance to achieving an optimal glucose control. As a result, the dynamics of insulin release from the pancreas are currently described by mathematical models that reproduce the behavior of the *β* cells using exclusively glucose levels and other hormones as inputs. To bridge this gap, we have combined, for the first time, metabolic and neural mathematical models in a unified system to reproduce to a great extent the ideal glucoregulation observed in healthy subjects. Our results satisfactorily replicate the CPIR and its impact during the post-absorptive phase. Furthermore, the proposed model gives insight into the physiological interaction between the brain and the pancreas in healthy people and suggests the potential of considering the neural information for restoring glucose control in people with diabetes.

**Graphical Abstract:**
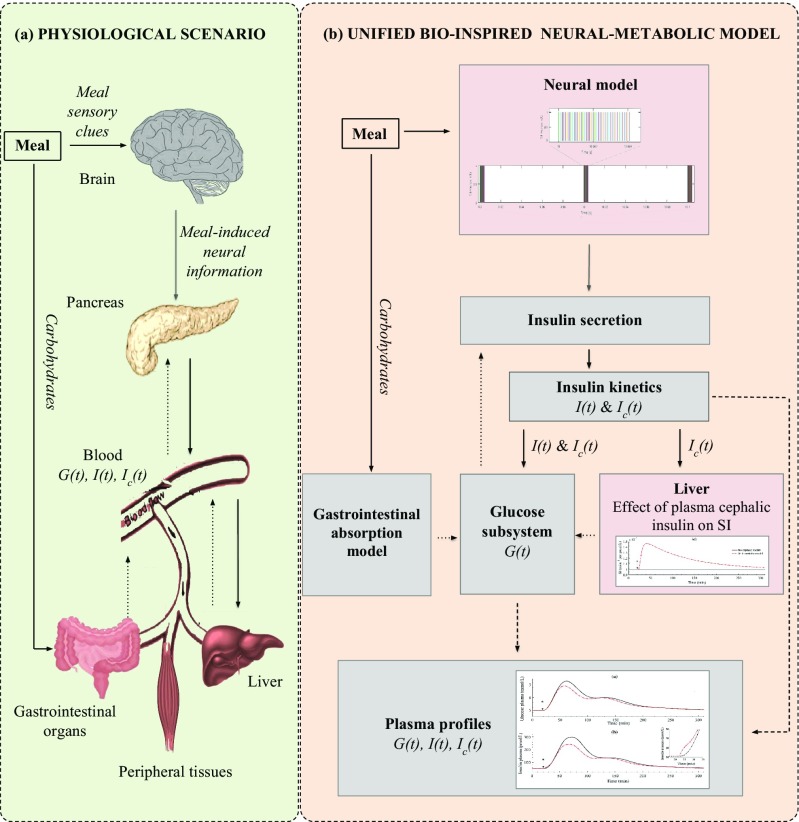
(a) Physiological scenario. Diagram of the biological interaction among the most important organs involved in glucose control during meal intake. (b) Scheme of the unified bio-inspired neural-metabolic model. Each of the boxes represents one subsystem of the model. The pink shades boxes depicts the novel subsystems introduced to the current metabolic models (grey shaded boxes). Insulin-related action and mass fluxes (solid black lines) and glucose-related action and mass flux (dotted black lines) are depicted to show the relationship among the blocks. *I*(*t*), *I*_*c*_(*t*), *G*(*t*) and *SI* related to plasma insulin, plasma cephalic insulin, plasma glucose and insulin sensitivity, respectively.

(a) Physiological scenario. Diagram of the biological interaction among the most important organs involved in glucose control during meal intake. (b) Scheme of the unified bio-inspired neural-metabolic model. Each of the boxes represents one subsystem of the model. The pink shades boxes depicts the novel subsystems introduced to the current metabolic models (grey shaded boxes). Insulin-related action and mass fluxes (solid black lines) and glucose-related action and mass flux (dotted black lines) are depicted to show the relationship among the blocks. *I*(*t*), *I*_*c*_(*t*), *G*(*t*) and *SI* related to plasma insulin, plasma cephalic insulin, plasma glucose and insulin sensitivity, respectively.

## Introduction

Maintaining robust control of glucose homeostasis is essential to guaranteeing the daily function of the human body through the provision of energy in cells via glycolysis. Therefore, to ensure a tight regulation of blood glucose fluctuations our bodies rely on the complex interaction of many organs, such as the pancreas and liver, acting through hormones and neurotransmitters. The brain has also been found to have a crucial role towards this objective [27, 36]. Evidence of its contribution on glucose regulation dates back to the work of the physiologist Claude Bernard, who for the first time showed a causal relationship between brain stimulation of the fourth ventricle in the hindbrain and an increase in plasma glucose levels [13]. Latter research in the field strengthened this evidence by bringing to light the mechanisms that underlie this neural control of glucose homeostasis [17, 27, 35, 41, 42]. However, these findings are just at the beginning of our understanding because most of the neural action schemes are still unknown [27].

Of remarkable interest has always been the study of the implications of central and peripheral neural mechanisms in regulating the endocrine pancreatic function [8, 25, 45, 56]. In particular, the nervous system has been shown to have a major role in the cephalic phase of insulin release (CIPR), which refers to the pre-absorptive secretion of insulin triggered by neural signals rather than to changes in plasma glucose concentrations after meal intake [3, 58].

**Fig. 1:**
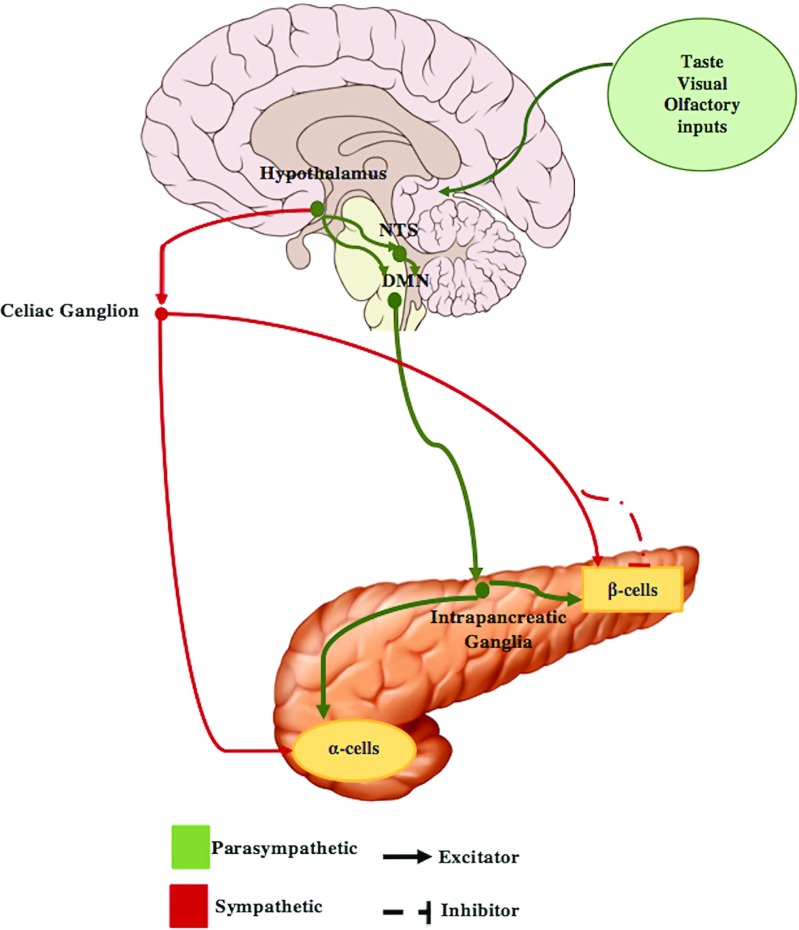
Diagram depicting the most important neural pathways to the pancreas during the cephalic phase

Between the two major pathways identified in regulating islet secretion, parasympathetic and sympathetic (see Fig. 1), only the former has been found to carry out a significant role in regulating the CPIR [1, 8, 32, 35, 42, 55]. The sympathetic innervation, on the contrary, is not likely to affect CPIR as it mainly inhibits insulin secretion in hypoglycaemia [4, 6, 8, 55]. There are two parasympathetic mechanisms which, through activation of the vagus nerve, enhance insulin and glucagon secretion: (i) cholinergic regulation via release of acetylcholine (ACh) and (ii) non-cholinergic mechanisms mediated by neuropeptides, such as vasoactive intestinal polypeptide (VIP), gastrin releasing peptide (GRP) and pituitary adenylate cyclase activating polypeptide (PACAP) [2, 14]. In some animals, like dogs, rats, and calves, the insulin secretion has been found to be mediated mainly by ACh because it was largely inhibited by atropine, which is a muscarinic antagonist [26]. On the contrary, in humans and pigs, there is evidence of other non-cholinergic neurotransmitters being implicated [3, 26]. The contribution of each parasympathetic mechanism to insulin secretion has been assessed with the use of the ganglionic blocker trimethaphan, which inhibits all the neural transmission to the pancreas [3]. However, the degree of involvement of non-cholinergic mechanisms to the hormonal secretion during the cephalic phase is still not clear [2, 8]. In addition, to the best of our knowledge, there are no reported data describing their release dynamics. Therefore, a mathematical model of the non-cholinergic signaling mechanisms is not available. On the contrary, models of the ACh release after vagal activation have been reported [22, 60]. For these reasons, only the cholinergic mechanism, i.e., through secretion of ACh, is included in the proposed model.

Regarding the characteristics of the CPIR, it lasts up to 10 min and has its peak within the first 4 or 5 min after the food ingestion [3, 47, 49, 52]. Its contribution to the entire postprandial insulin secretion is quite small, in the range of only the 1 to 3% of the total secretion (approximately 25% above the baseline levels) [47]. It is thought to act mainly on the hepatic glucose metabolism, by allowing a prompt inhibition of gluconeogenesis in the liver [3, 21, 34] and on the metabolism of fats by inhibiting lipolysis [26, 47]. This allows the body to prepare to the rapid and large increase in plasma glucose levels that occurs after meal intake. In addition, absence of the CPIR has been related to an impaired glucose tolerance after a meal through a reduction of postprandial hepatic glucose uptake [3, 21, 37, 46, 47]. Therefore, the CPIR affects the insulin sensitivity, which is a measure of the effectiveness of insulin action on the tissues. To further illustrate its importance, studies in people with type 2 diabetes (T2DM) with an impaired or absent CPIR have reported that injection of a small amount of insulin just after the meal intake resembling the cephalic insulin allowed to successfully increase glucose tolerance in these patients [3, 21, 47]. In conclusion, the resulting effect of the CPIR is a reduction of postprandial glycemic fluctuations and insulin secretion [3, 21, 26, 47].

It is worth noting that the cephalic insulin has not been found to directly affect glucose uptake by other peripheral organs [26].

Existing metabolic mathematical models are implemented using differential equations which describe the cellular dynamics for insulin release from the *β* cells using exclusively glucose levels and other metabolites as inputs [19, 29, 31]. On the other hand, mathematical models of the neuroregulation of many physiological processes have been developed, including modeling of the respiratory system [43], blood pressure [24], or heart rate among others [22]. However, to the best of our knowledge, models describing the effects of the neural regulation on the pancreatic function have not been reported. Hence, the important neural-pancreatic interaction is currently disregarded, although it is crucial to achieve an optimal glucose control.

To bridge this gap, we have combined for the first time metabolic and neural mathematical models in a unified physiological model to reproduce to a great extent the ideal glucoregulation seen in healthy subjects. This work motivates the development of more comprehensive models of the pancreatic secretion and encourage further investigations on the neural control of glucose homeostasis towards diabetes management.

## Methods

### The model

**Fig. 2:**
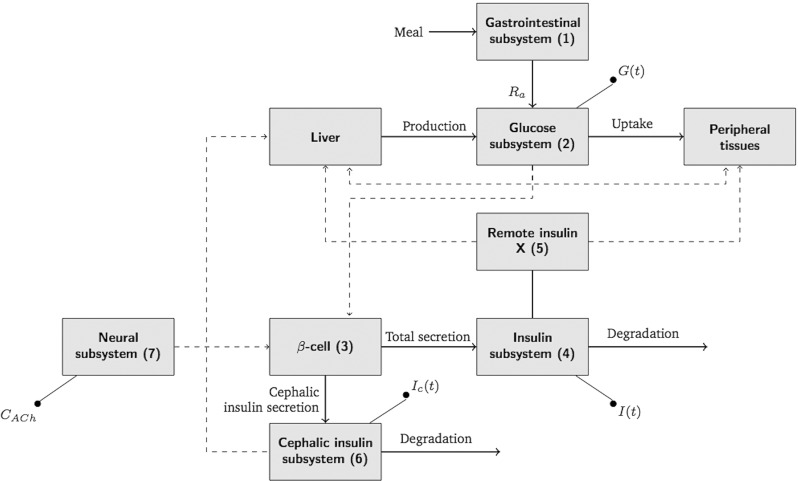
Diagram of the complete model showing the interaction regarding the mass fluxes (solid lines) and control actions (dashed lines) among the different subsystems (1–7). *I*(*t*), *I*_*c*_(*t*), and *G*(*t*)—plasma insulin, plasma cephalic insulin, and plasma glucose, respectively—are the outputs of each subsystem

Figure 2 depicts the complete scheme of the unified neural-glucose-insulin system model representing the fluxes of glucose and insulin and the control actions among them and the brain.

#### Gastrointestinal absorption model

The amount of carbohydrates taken during a meal is a key input for the glucose subsystem (see block 1 in Fig. 2). The gastrointestinal meal absorption model developed by Hovorka et al. was implemented [29, 31]. This model was selected for two main reasons: (i) its simplicity significantly reduces the number of parameters in comparison with other complex existing models [19], therefore facilitating the complete model identification, and (ii) it has been found to be sufficient to adjust the postprandial glucose profiles of standard meals like the one used in this work [29]. The output of this two-state model is the rate of plasma glucose appearance *R*_*a*_ (mg/min) after an ingestion of a certain amount of carbohydrates *D* (mg) in a meal. The model is defined by the following equations:

1 $$ \dot{F}(t) = \frac{1}{t_{maxG}}(-F(t) + A_{g}D \delta (t)), $$

2 $$ \dot{R_{a}}(t) = \frac{1}{t_{maxG}}(-R_{a}(t) + F(t)), $$

where *F(t)* denotes the glucose change in the first compartment, *t*_*maxG*_ (min) describes the time-to-maximum of CHO absorption, *A*_*g*_ (unitless) is the carbohydrate bioavailability, and *δ*(*t*) is a Dirac delta.

#### Glucose subsystem

The model of plasma glucose and insulin interaction developed by Bergman et al. [11] is used as the key subsystem for studying the interaction between insulin and glucose.

The model describes the whole body as a unique compartment with a basal concentration of glucose and insulin. The glucose production and disappearance is influenced by a remote insulin compartment, which is insulin dependent (see interaction among blocks 2 and 5 in Fig. 2).

The model is described by two differential equations:

3 $$ \dot{G}(t) = -[S_{G} + X(t)] G(t) + S_{G} G_{b} + \frac{R_{a}(t)}{V_{G}BW}, $$

4 $$ \dot{X}(t) = -p_{2}X(t) + p_{2}S_{I}[I(t) - I_{b}], $$

where *G(t)* is the plasma glucose concentration; *X(t)* is the remote active insulin effect on glucose uptake by the tissue and its uptake and production by the liver; *p*_2_ refers to the rate of degradation of the active insulin; *BW* (kg) is the subject’s body weight and *V*_*G*_ (dL/kg) is the glucose distribution volume.

The glucose effectiveness *S*_*G*_ (min^− 1^) is defined as the glucose ability *per se* to promote its own disposal and inhibit its production [29], i.e., independently of insulin. Insulin sensitivity *S*_*I*_ (min^−1^ per pmol/L) is defined as the ability of insulin to increase glucose effectiveness [29]. As a result, it characterizes the effect of insulin in the balance of glucose production and uptake.

#### Neural model: ACh secretion

To the extent of our knowledge, there is a lack of *in vivo* experiments in humans regarding the continuous secretion of ACh from the vagus nerve terminals in the endocrine pancreas after vagal stimulation. However, it is reasonable to assume that the kinetics of ACh secretion does not change from one nerve terminal to another. Therefore, in this first proposal, a model based on the ACh secretion by parasympathetic activation on the heart has been used [22, 60]. In particular, the model proposed by Dexter and colleagues [22] was selected.

As reported in the literature, basal insulin secretion is not affected by a blockade of the nervous signaling (e.g., by vagotomy or atropinization) [3, 26]. This is an indicator that there is not significant tonic cholinergic stimulation of the *β* cells in the fasting state. As a result, only the activation of the vagus nerve terminals elicited by food intake depletes a pool of ACh vesicles, where 0 ≤ *V* (*t*) ≤ 1 is the normalized quantity of ACh vesicles available for release (dimensionless). The change in ACh concentration in the interstitial space, which is represented by block 7 in Fig. 2, is given by:

5 $$\begin{array}{@{}rcl@{}} \dot{V}(t) &=& -\rho V(t)s(t) + K_{R} (1-V(t)) \text{ with } V(0)= 1, \end{array} $$

6 $$\begin{array}{@{}rcl@{}} R(t) &=& m\rho V(t)s(t), \end{array} $$

7 $$\begin{array}{@{}rcl@{}} \dot{C}_{ACh}(t) &=& -K_{D}C_{ACh}(t) + R(t) \text{ with } C_{ACh}(0)= 0,\end{array} $$

where *C*_*ACh*_(*t*) (nM) represents the interstitial concentration of ACh; *K*_*D*_ (min^-1^) is the rate of ACh enzymatic degradation; *s(t)* is the vagal firing patterns or stimulus; *K*_*R*_ (min^-1^) represents the rate of renewal of vesicles; *ρ* (unitless) is the fraction of the total vesicles (*V* ) released by each vagal stimulation; and *m* (nM) is the maximal concentration of ACh that can be released per stimulus.

The real physiological nervous stimulus consists of a burst of action potentials (APs) or spikes (0 or 1 events) with a certain firing frequency, i.e., inter spike time. In order to develop a detailed physiological model, in this work, the nervous stimulus has being modeled as a train of squared pulses (*s(t)* = 1 during the pulse and *s(t)* = 0 otherwise), each of them with a duration *δ* (ms) that resembles that of the real action potential (see Fig. 6). The activation of the train of pulses occurs in the instant the meal is taken *t*_*meal*_ (i.e., as soon as the food is ingested).

Following a first order Boltzmann dynamics, ACh concentration (nM) in the interstitial space eventually reaches a mean plateau value with time (with some superimposed ripple) that increases with increasing frequencies of stimulation. Only the value at the steady state reflects the action of the neural system, so a simplified version of the neural model considering only the mean concentration of ACh in the steady state was incorporated to the unified neural-metabolic model:

8 $$\begin{array}{@{}rcl@{}} V&=&\frac{K_{R}}{\rho \text{ } mean(s(t))+K_{R}}, \end{array} $$

9 $$\begin{array}{@{}rcl@{}} C_{ACh}&=&\frac{m \text{ } \rho \text{ } V \text{ } mean(s(t))}{K_{D}}.\end{array} $$

This simplified neural model solely depends on the firing frequency of the stimulus, since the higher the frequency, the higher the mean of the stimulus along time, leading to a higher steady-state value for ACh concentration (*C*_*ACh*_).

#### Insulin secretion

The model used for describing glucose-dependent insulin secretion is based on that proposed by Toffolo et al. [57] and Breda et al. [16], and reported by Dalla Man et al. [19] as:

10 $$\begin{array}{@{}rcl@{}} S(t) \!&=&\! \gamma I_{po}(t), \end{array} $$

11 $$\begin{array}{@{}rcl@{}} \dot{I}_{po}(t) \!&=&\! -\gamma I_{po}(t) + I_{p1}(t) + I_{p2}(t) \\ &&+S_{b} \text{ with } I_{po}(0) = I_{pob}, \end{array} $$

12 $$\begin{array}{@{}rcl@{}} I_{p1}(t) \!&=&\! \left\lbrace \begin{array}{ll} k_{Di} \dot{G}(t) & \text{if } \dot{G}>0,\\ 0 & \text{otherwise}, \end{array}\right. \end{array} $$

13 $$\begin{array}{@{}rcl@{}} \dot{I}_{p2}(t) \!&=&\! \left\lbrace\! \begin{array}{ll} {}-{}\alpha [I_{p2}(t)- \beta (G(t) - h)] & \!\text{if } \beta (G(t) - h)\! \geq -S_{b}\\ {}-{}\alpha [I_{p2}(t)+Sb] & \text{otherwise} \end{array}\ I_{p2}(0)= 0 \right.\\ \end{array} $$

where *S*(*t*) (pmol/kg/min) is the total glucose-dependent secreted insulin; *γ* (min^-1^) is the transfer rate constant between portal vein and liver *I*_*po*_(*t*) represents the amount of insulin in the portal vein, subindex *b* refers to the basal state; *I*_*p*1_(*t*) (pmol/kg) is responsible for the first phase of secretion, and *I*_*p*2_(*t*) (pmol/kg) characterises the slower second secretion phase; *k*_*Di*_ (pmol/kg per mg/dL) is the pancreatic responsivity to the glucose rate of change; *α* is the delay between glucose signal and insulin secretion; *β* (pmol/kg/min per mg/dL) is the pancreatic responsivity to glucose and *h* (mg/dL) is the threshold level of glucose above which the *β* cells initiate to produce new insulin. As stated in [19], *h* has been set to the basal glucose concentration (*G*_*b*_) to guarantee the steady state in basal conditions.

The pre-absorptive phase of insulin secretion from the *β* cell is dependent of the interstitial concentration of ACh. In addition, as the literature reports, there is a significant dependence on plasma glucose levels in the effects of ACh on insulin secretion [12, 26]. Both *in vitro* and *in vivo* studies have shown that the effect of ACh is present from 5-nM glucose levels (i.e., normal basal glucose levels) and its efficacy in insulin release significantly increases with higher glucose levels [12, 26]. Consequently, the novel equations defining ACh action on neurally mediated pre-absorptive insulin release has been defined as:

14 $$ \left\lbrace \begin{array}{ll} S_{c}(t) = \gamma I_{pc}(t), \\ \dot{I}_{pc}(t) = -\gamma I_{pc}(t) + Z(t), \end{array} \right. $$

15 $$ Z(t) = \left\lbrace \begin{array}{ll} K_{ACh}C_{ACh}(t)(G(t)-h) & \text{if } G(t)>h,\\ 0 & \text{otherwise}, \end{array} \right. $$

where *S*_*c*_(*t*) (pmol/kg/min) is the total amount of insulin secreted during the cephalic phase; *I*_*pc*_ represents the amount of cephalic insulin in the portal vein, with *I*_*pc*_(0) = 0; $\gamma (\min ^{-1}$) is again the transfer rate constant between portal vein and liver; and *K*_*ACh*_ (pmol/kg per nM per mg/dL) is the pancreatic responsivity to the ACh interstitial concentration.

As a result, the total amount of insulin secreted by the *β*-cells, *S*_*T*_(*t*) (pmol/kg/min), comprises the glucose-dependent insulin and the insulin elicited by neural stimulation:

16 $$ S_{T}(t) = S(t) + S_{c}(t). $$

No delay on the ACh action on cephalic insulin release was included in the proposed model because it was shown to be negligible during the simulations in the time scale of the kinetics of insulin secretion.

#### Insulin kinetics

The model of insulin kinetics that has been used is based on the two compartment model developed by Dalla Man and colleagues [19]. In this unified neural-metabolic model, it takes the total insulin secretion from the *β* cell (both glucose and ACh dependent insulin) as an input and determines the plasma insulin. In doing that, it takes into account the insulin degradation rate that occurs in both the liver and the periphery. The set of differential equations that describes this subsystem corresponds to block 4 in Fig. 2 and is the following:

17 $$ \left\lbrace\!\! \begin{array}{ll} \dot{I}_{l}(t) = -(m_{1} + m_{3}(t))I_{l}(t) + m_{2}I_{p}(t) + S_{T}(t) & I_{l}(0) = I_{lb},\\ \dot{I}_{p}(t) = -(m_{2} + m_{4})I_{p}(t) + m_{1}I_{l}(t) & I_{p}(0) = I_{pb},\\ I(t) = \frac{I_{p}}{V_{I}} & I(0) = I_{b}, \end{array} \right. $$

where *I*_*p*_ and *I*_*l*_ (pmol/kg) are insulin masses in plasma and in liver respectively; *I* (pmol/L) is the plasma insulin concentration; *I*_*b*_ represents the basal state; *S*_*T*_(*t*) (pmol/kg/min) is the total insulin secretion form *β*-cell; *V*_*I*_ (L/kg) is the insulin distribution volume; and *m*_1_,*m*_2_, and *m*_4_ (min^-1^) are rate parameters. The hepatic extraction of insulin *HE*, i.e., the insulin flux which leaves the liver irreversibly divided by the total insulin flux leaving the liver, is time varying [19]:

18 $$ HE(t) = -m_{5} S_{T}(t) + m_{6} \textit{with } HE(0) = 0, $$

and therefore:

19 $$ m_{3}(t) = \frac{HE(t) m_{1}}{1-HE(t)}. $$

An additional similar block of insulin kinetics but using solely the cephalic insulin secreted (see block 7 in Fig. 2) was included in order to have an independent quantification of its plasma concentration:

20 $$ \left\lbrace\!\! \begin{array}{ll} \dot{I}_{cl}(t) = -(m_{1} + m_{3}(t))I_{cl}(t) + m_{2}I_{cp}(t) + S_{c}(t) & I_{cl}(0) = 0,\\ \dot{I}_{cp}(t) = -(m_{2} + m_{4})I_{cp}(t) + m_{1}I_{cl}(t) & I_{cp}(0) = 0,\\ I_{c}(t) = \frac{I_{cp}}{V_{I}} & I_{c}(0) = 0. \end{array} \right. $$

In this way, an independent effect of the part of the total plasma insulin correspondent to the CPIR in the glucose hepatic production can be achieved.

#### Unified neural-metabolic model: effect of cephalic released insulin

The cephalic insulin acts on the liver causing a prompt inhibition of gluconeogenesis and enhancing the action of postprandial insulin on the hepatic glucose uptake, therefore increasing insulin sensitivity (see Fig. 2) [3, 21, 37, 46]. Hence, in this model the insulin sensitivity changes over time based on the concentrations of the cephalic insulin in plasma *I*_*c*_(*t*) (pmol/L):

21 $$ \dot{S_{I}}(t) = -p_{4} S_{I}(t) + p_{4}(S_{Ib} + k_{S_{I}}I_{c}(t)) \\ S_{I}(0) = S_{Ib}, $$

where *S*_*Ib*_ (min^–1^ per pmol/L) is the basal insulin sensitivity, set to its value when there is no neural control (i.e., *I*_*c*_(*t*) = 0), $k_{S_{I}}$ ($\frac {L ~ kg}{pmol^{2}})$ defines the action of the cephalic plasma insulin and *p*_4_ (min^-1^) is the rate constant describing the dynamics of glucose hepatic production inhibition.

### Experimental data

**Fig. 3:**
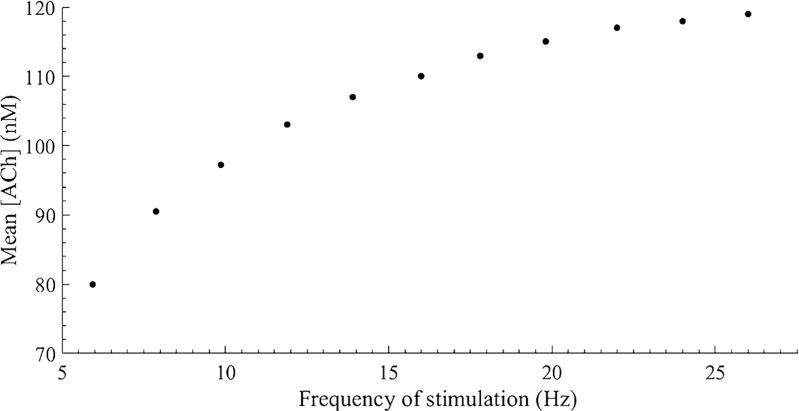
Mean interstitial concentration of acetylcholine (nM) over time

Experimental data found in the literature were employed for the parameters identification task. The data were extracted from the graphs reported in literature using the software ScanIt [5]. For the identification of the ACh release subsystem, the results obtained by Dexter et al. [22] in a study of the changes in heart rate elicited by vagal stimulation were used (see Fig. 3).

**Fig. 4:**
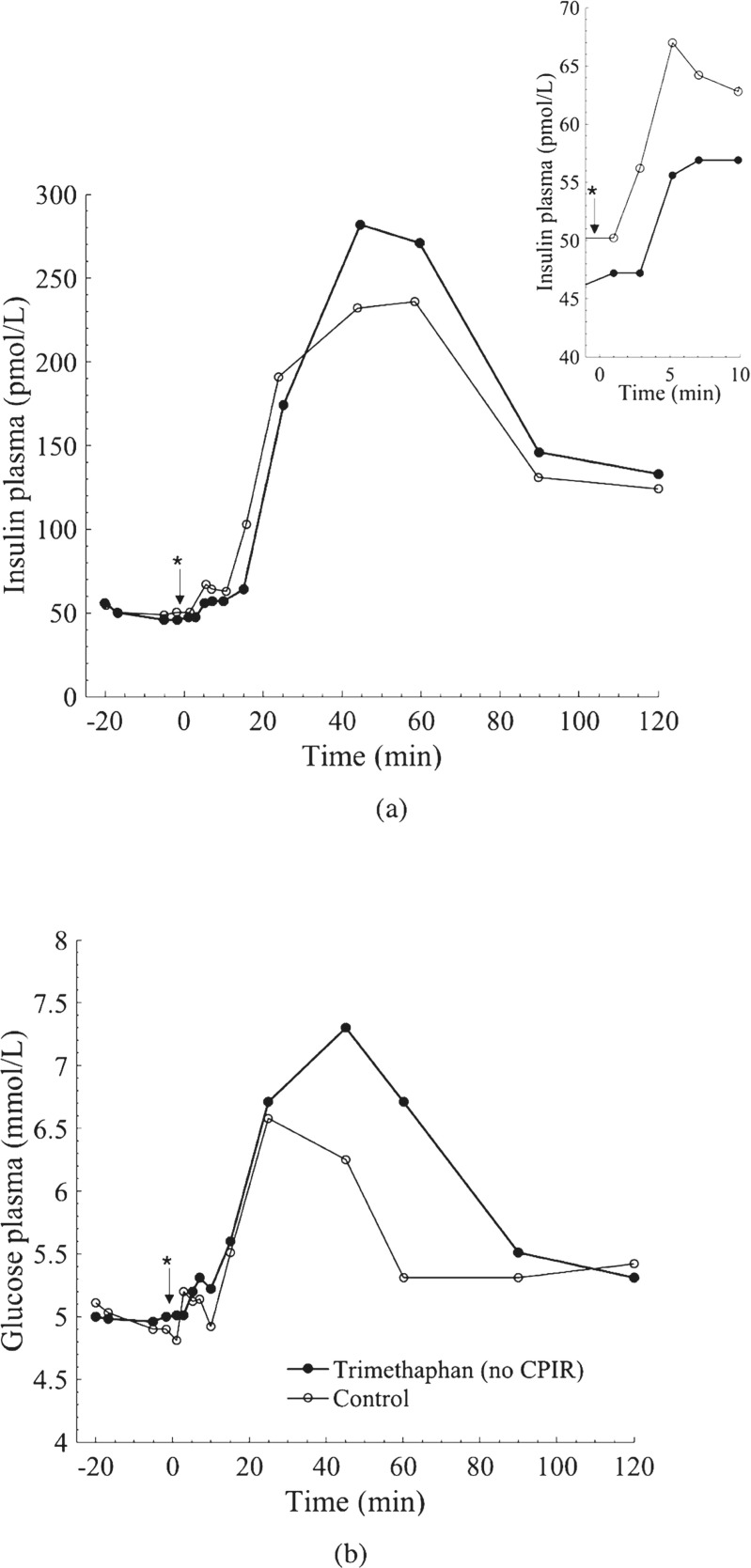
Average serum insulin and plasma glucose levels (N = 6). **a** Insulin profile. The small insert shows the insulin levels 10 min after meal intake (depicted with *), which corresponds to the cephalic period. **b** Glucose profile. At time 0 the meal was served

For the rest of the subsystems, the mean results of six female healthy subjects in presence and absence of the ganglionic blocker trimethaphan reported by Ahrén et al. [3] were used (see Fig. 4). The presence of trimethaphan allows achieving a purely metabolic system without any neural control, whereas in its absence, the neural contribution is also shown.

### Parameter identification

**Fig. 5:**
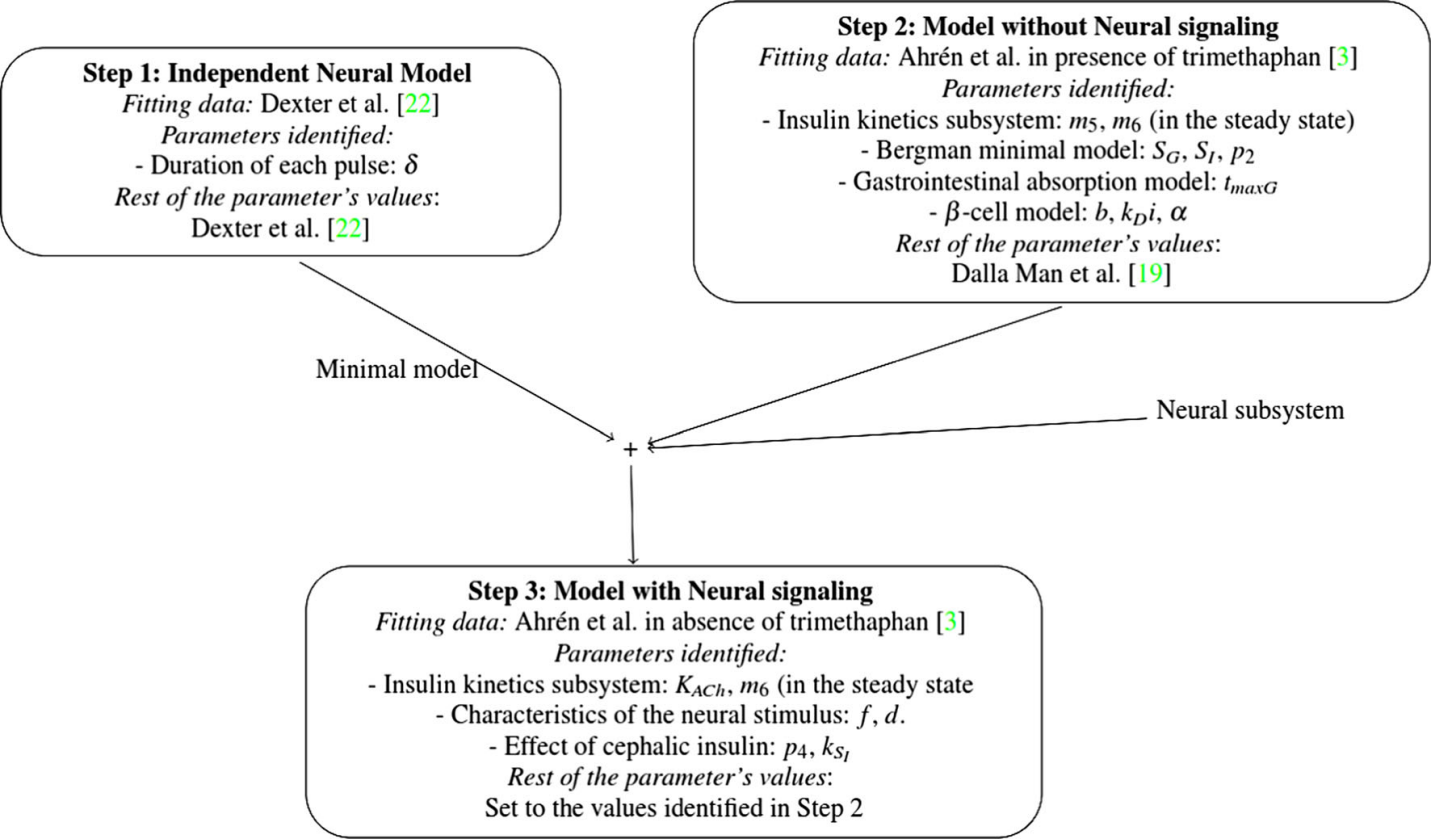
Summary of the steps carried out for the parameters identification task

A sensitivity analysis was carried out to reduce the number of simultaneously identified parameters. Those that had the lowest effect to the system were fixed to their value reported in existing populational models (see Table 2) [19, 22]. In addition, the parameters related with the ACh secretion were fixed to the values defined by Dexter et al. [22] to be consistent with the hypothesis of similar kinetics of release from different vagus nerve terminals. To further reduce the of degrees of freedom and to develop a consistent model, the parameters of each subsystem were progressively identified in three steps by minimizing the coefficient of variation (CV), calculated as the ratio of the root mean squared error (RMSE) to the mean of the dependent variable. An Evolution Strategy with Covariance Matrix Adaptation (CMA-ES) algorithm for nonlinear global optimization was employed [7, 28]. Figure 5 summarizes the identification process and the overall integration of the model.

During the optimization process, the parameters were constrained to lie within a range of values with (i) physiological significance and (ii) lie in the range of values reported in the literature, as shown in Table 1. The final estimation values of the parameters can be found in Table 2. The models were numerically integrated using a RK4 integrator with a fixed integration step size of 0.1 ms for the neural model and 1 min for the unified neural-metabolic model.

**Table 1:** Constraints used in the parameter identification process and their explanation

Process	Parameter	Constraints	Units	Explanation
Neural model of ACh release	*δ*	4.2	*ms*	Absolute refractory period of APs is 1 ms and the maximum duration 5 ms [33]
Bergman minimal model (extended)	*p*_2_	0.01–0.1	*m* *i* *n* ^− 1^	Covers the great variability of values reported in the literature [20, 40, 59]
	*S*_*Ib*_	(0.66 − 6.79) ⋅ 10^− 4^	*min*^− 1^ per *μmol*/*mL*	Reported by Bergman et al. [11]
	*S*_*G*_	0.002-0.039	*m* *i* *n* ^− 1^	Covers the range reported by Dalla Man et al. [20] and Steil et al. [44]
Gastrointestinal absorption model	$t_{\max G}$	35–45	*min*	Cover the value reported by Hovorka et al. [31]
Insulin secretion	*k*_*Di*_	1–3	*pmol*/*kg* per *mg*/*dL*	Covers the values reported by Dalla Man et al. [19]
	*α*	0.01–0.5	*m* *i* *n* ^− 1^	Covers the values reported by Dalla Man et al. [19]
	*β*	0.1–0.2	*pmol*/*kg* per *mg*/*dL*	Covers the values reported by Dalla Man et al. [19]
Insulin kinetics	*m*_5_	0.015–0.025	*minkg*/*pmol*	Covers the values reported by by Dalla Man et al. [19] and to ensure stability in the steady state
	*m*_6_	0.6–0.7	*minkg*/*pmol*	Covers the values reported by by Dalla Man et al. [19] and to ensure stability in the steady state
Neural stimulus	d	0.5–3	*min*	No literature found.
	f	0.25–25	*Hz*	Range reported by Holst et al. [30]
Cephalic insulin release	*K*_*ACh*_	0.1–5	*pmol*/*kg* per *nM*	–
Action of cephalic insulin	*p*_4_	0.005–0.5	*m* *i* *n* ^− 1^	–
	$k_{S_{I}}$	1 ⋅ 10^− 5^ − 0.1	$\frac {L ~ kg}{pmol^{2}}$	–

## Results

### Independent neural model identification

**Fig. 6:**
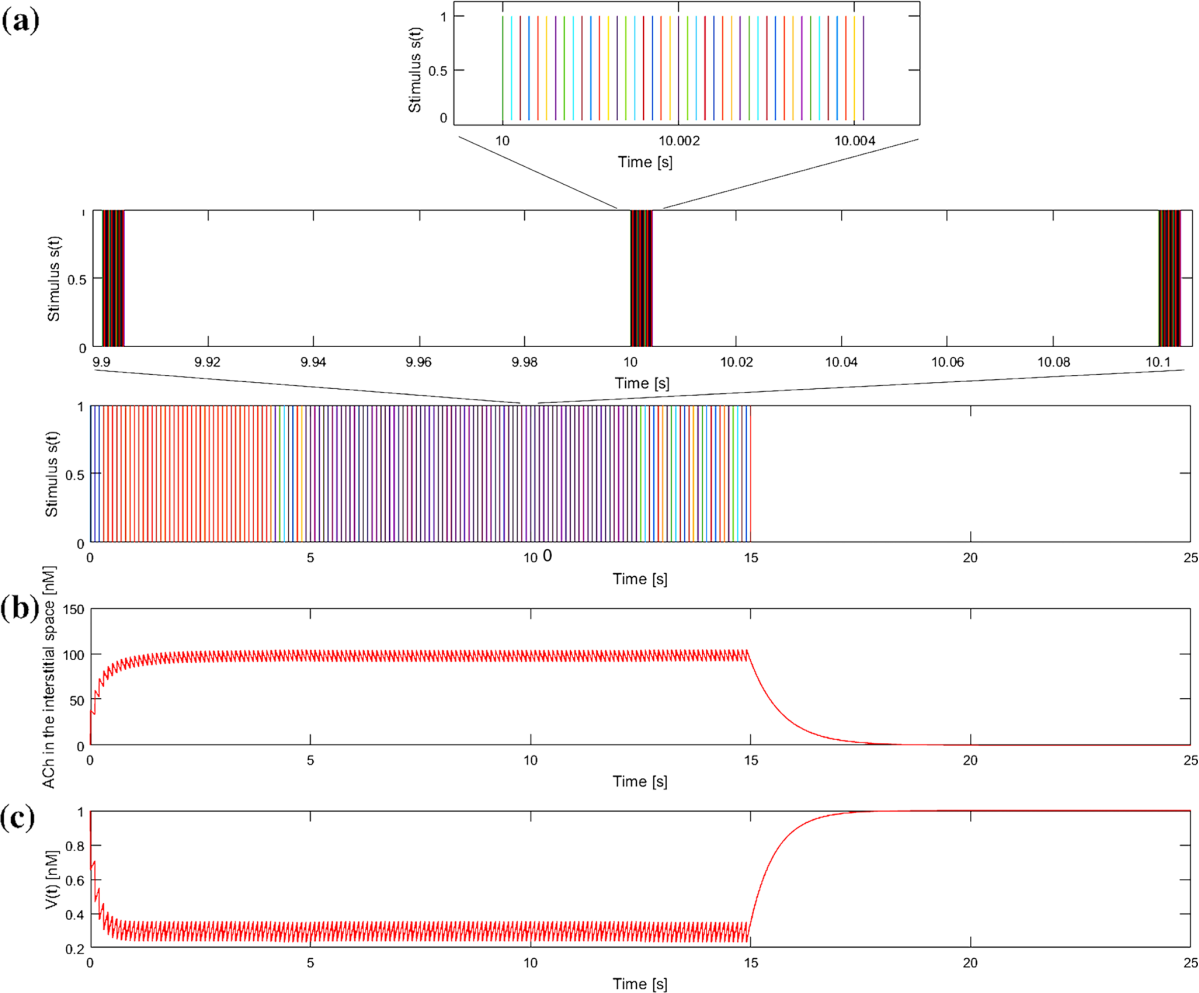
Neural model of ACh release. **a** Neural stimulus of 10 Hz and 15 s of overall duration and zoom to visualize the train of pulses and its discretization. **b** Profile of ACh concentration in the interstitial space. **c** Profile of vesicles secretion (V(t)) from the nerve terminals

The values of the parameters of the independent neural model, including the duration of the pulses identified in the validation process are reported in Table 2. Figure 6 depicts the profiles of vesicles secretion and ACh concentration in the interstitial space, together with the correspondent stimulus, achieved with the independent neural model. To improve the visualisation of the results, an exemplary train of pulses (stimulus) with a firing frequency of 10 Hz and a total duration of the train of pulses of 15 s was modeled. It can be clearly seen how the concentration of ACh in the interstitial space increases until reaching a plateau level with some ripple as long as the stimulus was present and decreases exponentially afterwards.

**Table 2:** Model parameters

Process	Parameter	Value	Units
Neural model of ACh release	*K*_*D*_	0.00139^†^	*m* *i* *n* ^− 1^
	*K*_*R*_	0.0035^†^	*m* *i* *n* ^− 1^
	*ρ*	0.6^†^	*D* *i* *m* *e* *n* *s* *i* *o* *n* *l* *e* *s* *s*
	*m*	55^†^	*n* *M*
	*δ*	4.2	*ms*
Bergman minimal model (meal extended)	*p*_2_	0.094	*m* *i* *n* ^− 1^
	*S*_*Ib*_	6 ⋅ 10^− 4^	*min*^− 1^ per *μmol*/*mL*
	*S*_*G*_	0.0104	*m* *i* *n* ^− 1^
	*V*_*G*_	1.88*	*dL*/*kg*
Gastrointestinal absorption model	*A*_*g*_	0.85^⋆^	Dimensionless
	*t*_*maxG*_	40	$\min $
Insulin secretion	*γ*	0.5*	*m* *i* *n* ^− 1^
	*k*_*Di*_	1.54	*pmol*/*kg* per *mg*/*dL*
	*α*	0.29	*m* *i* *n* ^− 1^
	*β*	0.18	*pmol*/*kg* per *mg*/*dL*
Insulin kinetics	*V*_*I*_	0.05*	*L*/*kg*
	*m*_1_	0.19*	*m* *i* *n* ^− 1^
	*m*_2_	0.48*	*m* *i* *n* ^− 1^
	*m*_4_	0.194*	*m* *i* *n* ^− 1^
	*m*_5_	0.023	*minkg*/*pmol*
	*m*_6_	0.67	*Dimensionless*
	*H* *E*_*b*_	0.6*	*Dimensionless*
Neural stimulus	d	0.96	*min*
	f	20	*Hz*
Cephalic insulin release (cephalic phase)	*K*_*ACh*_	3.17	*pmol*/*kg* per *nM*
Action of cephalic insulin	*p*_4_	0.01	*m* *i* *n* ^− 1^
	$k_{S_{I}}$	9.3 ⋅ 10^− 5^	$\frac {L ~kg}{pmol^{2}}$

* Values taken from [19], ^†^values taken from [22], ^⋆^values taken from [31]

### Complete model identification

**Fig. 7:**
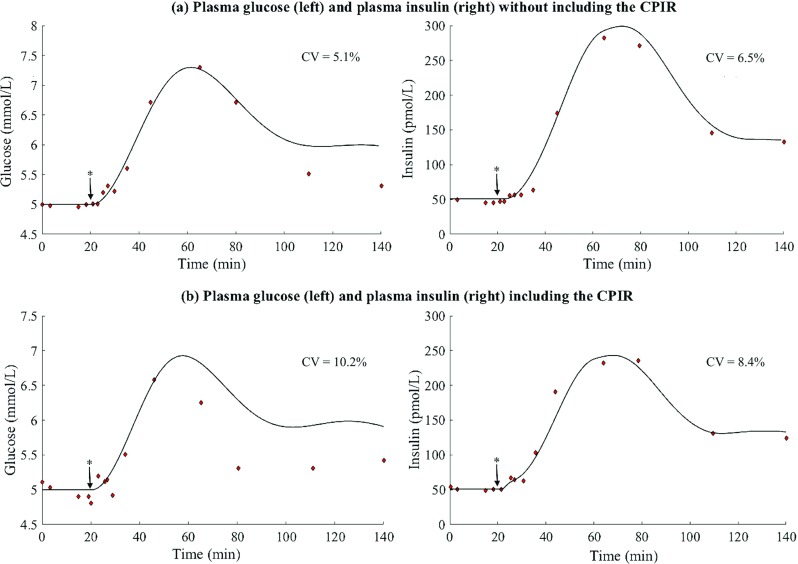
Comparison of the results of the fitted model with the experimental data. **a** Metabolic model without neural control. **b** Metabolic model including the neural control. Left: Glucose profile. Right: Insulin profile. Model results (continuous line) and fitting data set (red points). The model parameters reported in Table 2 were used. Meal time (*), occurs 20 min after the beginning of the simulation

All the parameters identified for each biological process can be found in Table 2. Figure 7 shows graphically the fitting of the model in absence (Fig. 7a) and presence (Fig. 7b) of the neural control to the corresponding experimental data. The quality of the fitting regarding the glucose profiles is CV = 10.2% and CV = 5.1% in presence and absence of CPIR, respectively. For the profiles of plasma insulin the obtained fitting errors are CV = 8.4% and CV = 6.5% in presence and absence of CPIR, respectively.

### Impact of the CIPR

**Fig. 8:**
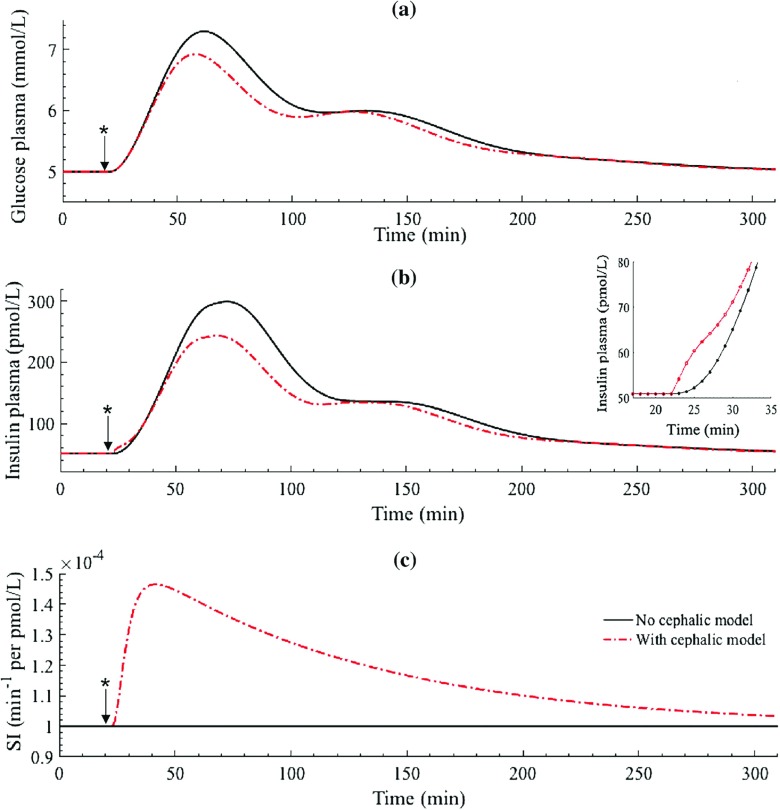
Postprandial glucose, insulin, and insulin sensitivity profiles along time in presence and absence of the cephalic phase. **a** Glucose profile. **b** Insulin profile. The small insert depicts the insulin levels corresponding to the cephalic phase (from the ingestion of the meal to 15 min after it). **c** Insulin sensitivity profile. The meal (*) was given 20 min after the beginning of the simulations

A comparison between the postprandial glucose and insulin profiles obtained in presence and absence of the neural contribution is depicted in Fig. 8. The temporal evolution of the insulin sensitivity as a result of the neural control is depicted as well, showing a peak at 17 min after meal intake and progressively decreasing with time afterwards. The amount of plasma insulin secreted during the cephalic phase (incremental area under the curve *iAUC*_*CPIR*_) corresponds to a 0.84% of the total plasma insulin secreted in the considered interval (from 0 to 140 min).

Table 3 presents the iAUC of the insulin and glucose profiles obtained in presence and absence of the neural contribution and reports the percentage of reduction of the postprandial plasma levels (19.6% and 24.6% of reduction for postprandial levels of plasma glucose and insulin respectively).

## Discussion

The proposed model closely replicates the characteristics of the CPIR reported in literature. Firstly, the peak of cephalic insulin release shows a maximum at 5.5 min after meal ingestion, which is within the interval of 3–6 min previously reported in experimental studies [47, 49, 52]. Secondly, it represents approximately 0.84% of the total insulin secreted, which is slightly lower than the previously reported range of 1 ± 3 % [47]. However, it is in agreement with the values reported by Ahrén et al. (0.80 ± 0.22*%*) [3], whose experimental results were used for the model’s identification. In addition, the insulin sensitivity shows a maximum at 17 min after meal intake due to the enhanced action of insulin on hepatic glucose uptake, being also consistent with previous research [3, 37]. From Fig. 8 and Table 3, it can be seen that inclusion of the small amount of neurally mediated pre-absorptive insulin has a powerful and essential effect in reducing postprandial plasma glucose and insulin levels after the meal intake. This outcome describes the causal inverse relationship between the neurally mediated pre-absorptive insulin and the postprandial hyperglycemia and hyperinsulinemia reported by previous studies [3, 21, 47, 53]. As a result, the proposed neural model of pre-absorptive insulin secretion seems to capture well the physiological effect of the cephalic insulin on hepatic glucose metabolism.

**Table 3:** Relative decrease in postprandial glucose and insulin plasma levels for each cephalic model with respect to the absence of neural control

	Without neural control	With neural control	% of reduction
iAUC postprandial plasma insulin	15.4 nmol/L	11.6 nmol/L	24.6%
iAUC postprandial plasma glucose	174.8 mmol/L	140.1 mmol/L	19.6%

However, the lack of knowledge regarding the neural stimulation of the endocrine pancreas hinders the development of comprehensive mathematical models of its behavior. In this study, we assumed that the vagus nerve’s terminals in different organs have similar kinetics of ACh secretion. Despite appearing to be numerically valid because it allows an accurate representation of the characteristics of the CPIR, it does not represent the real neural-pancreatic interaction. In addition, the CIPR on humans is highly dependent on the type, intensity, and duration of the stimuli [9, 48, 51]. Therefore, further experimental research is needed to identify the characteristics of the nervous signals to the pancreas and their relation with the meal type and size [27].

Moreover, scarcity of large and complete experimental data sets complicates the development of an accurate model in some aspects. Firstly, experimental studies have reported high variability in the pre-absorptive phase of insulin secretion among individuals [10, 47]. Consequently, the mean profile of only six healthy subjects will probably not reflect this intra-subject variability. Secondly, the degrees of freedom of the model was very large to allow an adequate identification of the parameters. Even though the step-wise strategy was implemented to reduce the number of simultaneously estimated parameters, it is well known that modeling with insufficient data generally leads to over-fitting, and therefore poor generalization. Hence, experimental data describing each subsystem in the presence and absence of neural regulation is needed to accurately identify and validate this model.

Finally, the neural control of the pancreatic secretion was modeled by considering the cholinergic signaling mechanisms. Disregarding the non-cholinergic pathways might also account for some uncertainties in the results. However, the degree of uncertainty is unclear because of the discrepancies found in the literature regarding the impact of atropine on the inhibition of the CPIR. In fact, some studies report an effective action [15, 50]; whereas in others, its effect is lower [3]. These variations have been explained by the use of different experimental conditions, types of food stimuli and gender of subjects (men have been shown to have a higher sensitivity to atropine [3, 50]).

All these limitations might explain the deviations of the model from the experimental data during the postprandial phase, especially regarding the plasma glucose profiles (see left panels in Fig. 7a–b). Despite them, the unified neural-metabolic successfully reproduce the characteristics of the CPIR and gives insight into the dynamics of its postprandial effects. This finding, while preliminary, suggests that existing models of glucose homeostasis, which currently neglect the neural contribution, would benefit from acquiring a better understanding of the physiological basis of the interaction between the brain and the pancreas.

## Conclusion

To the best of our knowledge, this work is the first attempt at defining a physiological neurally mediated metabolic model in healthy subjects. In it, the pancreas secretion is no longer controlled solely by hormones and metabolites, as current models do, but also includes the essential effect of the neural control in achieving an optimal glucose control.

Notwithstanding the limitations of the proposed model, the results closely represents the physiological effect of the cephalic insulin, as well as the glucose and insulin profiles in the presence of the neural innervation observed in healthy people. Hence, it presents a more detailed physiological model of the complex regulation of the healthy endocrine pancreas. This is important as *in silico* models provide a good platform to optimize systems and provide insight without the need for initial clinical validation on animals [23, 38].

In addition, it lays the foundation for the development of physiological models that reproduce to a great extent the complex regulation of glucose homeostasis in people with diabetes. This will allow existing simulators of diabetes mellitus to provide improved environments for testing treatments and monitoring interventions [18, 54]. It also opens the door to new approaches for the design of a bio-inspired artificial pancreas for type 1 diabetes treatment [29, 39], by completely closing the controlling loop using the neural signals to inform the controller about meal ingestion information.

In conclusion, the proposed work gives insight into the physiological basis of the nervous control of the pancreatic secretion and suggests the potential benefit of considering the neural information for restoring glucose control in people with diabetes.
